# Multifunctional 3D-Printed Polylactic Acid/Hydroxyapatite Systems for Cranial Applications: Functionalization and Local Anti-Inflammatory Drug Delivery

**DOI:** 10.3390/polym18131608

**Published:** 2026-06-28

**Authors:** Alessia D’Andrea, Sara Biesuz, Elena Mazzinelli, Giuseppina Nocca, Ilaria Cacciotti

**Affiliations:** 1 Department of Engineering, University of Rome Niccolò Cusano, Via Don Carlo Gnocchi 3, 00166 Rome, Italy; dandrea.alessia.14@gmail.com (A.D.); sara.biesuz@unicusano.it (S.B.); 2 Dipartimento di Scienze Biotecnologiche di Base, Cliniche Intensivologiche e Perioperatorie, Università Cattolica del Sacro Cuore, Largo Francesco Vito 1, 00168 Rome, Italy; elena.mazzinelli@unicatt.it (E.M.); giuseppina.nocca@unicatt.it (G.N.); 3 Fondazione Policlinico Universitario “A. Gemelli”, IRCCS, Largo Agostino Gemelli 8, 00168 Rome, Italy

**Keywords:** polylactic acid, hydroxyapatite, dexamethasone, drug delivery, cranial implants

## Abstract

Traumatic Brain Injuries (TBIs) frequently require cranioplasty procedures to restore skull integrity and protect underlying brain. Conventional cranial implants are often limited by inadequate osteointegration, risk of inflammation, infection, or the need for secondary surgical interventions. In this study, a multifunctional strategy for cranial reconstruction is proposed, combining additive manufacturing, bioactive surface functionalization, and local drug delivery. Porous polylactic acid (PLA) scaffolds were fabricated by Fused Deposition Modelling (FDM) to obtain lightweight structures with controlled porosity. The scaffolds were subsequently functionalized with hydroxyapatite coatings, deposited through sol–gel, to provide osteointegrative properties. To locally modulate post-implant inflammatory responses, a drug delivery system based on polycaprolactone (PCL) microparticles loaded with dexamethasone was developed and entrapped within hydroxyapatite-coated PLA structures. The produced systems were extensively characterized in terms of morphology, mechanical and thermal behavior, structural properties, biological response, and drug release behavior. Results demonstrated that the 3D-printed scaffolds exhibited homogeneous hydroxyapatite coatings, whose continuity and retention were enhanced by NaOH surface pre-treatment. Biological assays demonstrated that HAp coating significantly improved cell viability and osteogenic differentiation, confirming the osteoconductive potential of the scaffolds for craniofacial bone regeneration applications. Dexamethasone-loaded PCL microparticles were successfully integrated into the coated scaffolds, exhibiting controlled drug release, absence of cytotoxicity, and homogeneous distribution within the porous architecture, thereby demonstrating the feasibility of a multifunctional platform combining bone-regenerative and therapeutic delivery functionalities. Overall, the proposed multifunctional scaffolds represent a promising, low-cost and customizable approach for advanced cranioplasty applications, integrating structural support, osteointegration and local anti-inflammatory therapy within a single system.

## 1. Introduction

Traumatic Brain Injuries (TBIs) represent a major global health issue, frequently leading to cranial defects that require surgical reconstruction to restore skull integrity and protect the underlying neural tissue. Cranioplasty is therefore a crucial procedure not only for mechanical protection and aesthetic restoration, but also for improving cerebral hemodynamics and neurological function [1,2,3]. Despite significant advances in surgical techniques, the ideal cranial implant has not yet been identified, as currently adopted solutions still present limitations related to osteointegration, inflammatory response, infection risk and long-term clinical performance [4,5].

Traditional inert implants, including metallic and polymeric devices, mainly provide structural support but lack the ability to actively interact with the surrounding biological environment. Conversely, bioactive ceramic implants, such as hydroxyapatite-based systems, closely resemble the mineral phase of bone and promote osteointegration, yet often suffer from inadequate mechanical properties, especially when porosity is introduced to enhance biological performance [6,7,8]. As a result, increasing attention has been directed toward hybrid strategies that combine the mechanical reliability of polymers with the biological activity of ceramics [9,10].

In this context, additive manufacturing has emerged as a powerful tool for biomedical applications, enabling the fabrication of patient-specific implants with controlled geometry and internal architecture [11,12,13,14]. Among the available techniques, Fused Deposition Modelling (FDM) offers significant advantages in terms of cost-effectiveness, accessibility and versatility, making it particularly attractive for the production of personalized cranial scaffolds [15,16]. Polylactic acid (PLA), a biodegradable and biocompatible polymer approved for medical use, has been widely investigated for bone tissue engineering due to its favorable mechanical properties and ease of processing [17,18]. Nevertheless, the intrinsic bioinert nature of PLA limits direct bone bonding, thereby requiring surface functionalization to enhance osteogenic potential [19,20].

Surface modification of polymeric scaffolds with calcium phosphate-based coatings, particularly hydroxyapatite (HAp), represents an effective strategy to improve osteoinductivity while preserving the mechanical integrity of the underlying structure [21,22]. HAp coatings have been shown to promote cell adhesion, proliferation and differentiation, facilitating bone ingrowth and long-term implant integration [23,24]. However, osteointegration alone is often insufficient to ensure optimal clinical outcomes, as post-surgical inflammation and associated complications remain significant challenges in cranioplasty procedures [25,26].

To address these issues, recent research has increasingly focused on the development of multifunctional implants capable of simultaneously providing mechanical support, biological stimulation and therapeutic action. In particular, the incorporation of Drug Delivery Systems (DDSs) into polymeric scaffolds has gained attention as a strategy to locally modulate inflammatory responses while reducing systemic side effects [27,28]. Biodegradable polymeric microparticles, especially those based on polycaprolactone (PCL), offer sustained and controllable drug release profiles, making them suitable carriers for anti-inflammatory agents in bone regeneration applications [29,30]. Dexamethasone, a well-established glucocorticoid, has been widely investigated for its anti-inflammatory efficacy in tissue engineering and local delivery systems [31,32].

The integration of osteoinductive surface coatings and local anti-inflammatory drug delivery within a single 3D-printed scaffold therefore represents a promising yet still underexplored approach for advanced cranioplasty applications. By combining structural design, bioactivity and therapeutic functionality, such multifunctional systems have the potential to overcome the limitations of conventional implants and improve long-term clinical performance.

The aim of this study is to develop and characterize a multifunctional cranial scaffold produced by additive manufacturing, integrating structural support, osteoinductive surface functionalization and local anti-inflammatory drug delivery within a single implant. In particular, the proposed system combines: (i) a patient-customizable FDM-manufactured PLA scaffold providing structural support, (ii) a sol–gel-derived hydroxyapatite coating aimed at enhancing osteointegration, and (iii) dexamethasone-loaded PCL microparticles designed for local anti-inflammatory therapy.

While these strategies have been separately reported in the literature, studies integrating osteoconductive surface functionalization and local anti-inflammatory drug delivery within the same 3D-printed cranial scaffold remain limited. Therefore, the innovation of the present work lies in the development and preliminary validation of a multifunctional cranial reconstruction platform capable of simultaneously addressing structural, biological, and therapeutic requirements.

Specifically, porous PLA scaffolds were fabricated by Fused Deposition Modelling to obtain lightweight structures with controlled architecture suitable for cranial applications. The scaffolds were subsequently functionalized with hydroxyapatite coatings, deposited through different approaches, in order to enhance osteoinductive properties without compromising mechanical performance. In parallel, a Drug Delivery System based on biodegradable PCL microparticles loaded with dexamethasone was developed to locally modulate post-implant inflammatory responses.

The proposed systems were systematically characterized in terms of morphological, mechanical, thermal and structural properties, as well as biological response and drug release behavior, with the objective of assessing its suitability as an advanced, low-cost and customizable solution for cranioplasty applications.

## 2. Materials and Methods

### 2.1. Fabrication of 3D-Printed PLA Scaffolds

Porous scaffolds were designed using Autodesk Fusion 360 (version 2604.1.48) and processed for printing using Ultimaker Cura (version 5.6.0).

**Figure 1:**
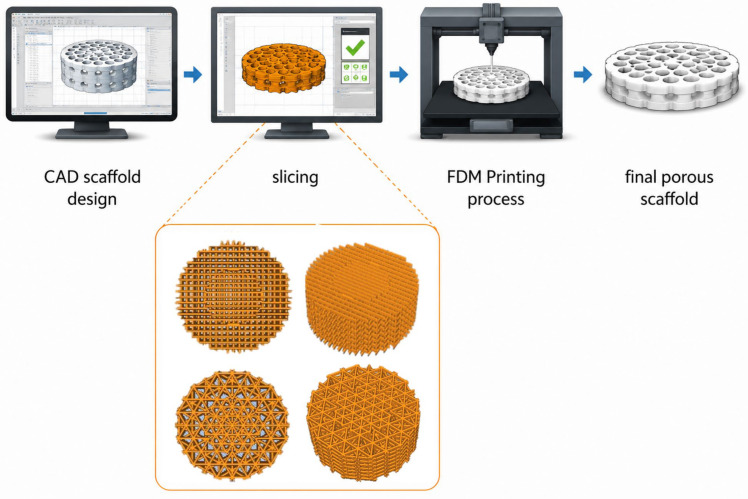
Schematic representation of the fabrication workflow of the porous PLA scaffold by fused deposition modelling (FDM), including CAD scaffold design, slicing (showing representative infill patterns: linear and triangular, in planar and 3D views), FDM printing process and final porous scaffold.

The selected scaffold design consisted of a cylindrical structure (diameter 10 mm, height 6 mm) characterized by a hierarchical porous architecture comprising an outer cylindrical region and an inner cylindrical core with differentiated pore dimensions, as schematized in Figure 1. This design was selected to promote cell infiltration, nutrient transport, and tissue ingrowth while maintaining adequate structural stability for cranial applications.

Scaffold fabrication was performed by Fused Deposition Modelling (FDM) using a commercially available white PLA filament (printing temperature range 170–210 °C, FILOALFA, Torino, Italy) (Figure 1). Printing was carried out using an Ender-3 Pro 3D printer (Creality, Shenzhen Creality 3D Technology Co., Ltd., Shenzhen, China) equipped with a 0.2 mm nozzle.

During the design phase, the scaffold geometry was kept constant, while different infill patterns were initially investigated to evaluate their suitability in terms of structural integrity, manufacturability and compatibility with subsequent surface functionalization and drug delivery integration. In particular, two infill patterns were considered, namely linear (L) and triangular (T). Based on these preliminary assessments, a final scaffold configuration was selected and used for all experiments presented in this study. The selected design consisted of a cylindrical scaffold geometry with a hierarchical porous architecture, combining interconnected macroporosity with smaller-scale porosity through differentiated inner and outer cylindrical regions. This configuration was chosen to promote nutrient diffusion, cellular infiltration and tissue ingrowth, while maintaining adequate mechanical stability for cranial applications. Printing parameters were optimized to ensure reproducibility and dimensional accuracy of the fabricated scaffolds. The main FDM printing parameters adopted in this study are summarized in Table 1. The reported printing parameters were selected based on a combination of manufacturer recommendations for the employed PLA filament, preliminary printing trials, and previous literature concerning FDM fabrication of porous PLA scaffolds for bone tissue engineering applications [19,20,33]. Particular attention was devoted to achieving dimensional accuracy, reproducibility of the porous architecture, adequate interlayer adhesion, and preservation of pore interconnectivity. Parameters such as printing temperature, layer height, print speed, and infill configuration were optimized through iterative preliminary tests to obtain stable structures without printing defects while maintaining the designed pore geometry. After fabrication, the scaffolds were visually inspected and stored under dry conditions prior to further processing.

### 2.2. Hydroxyapatite Coating of PLA Scaffolds

Prior to hydroxyapatite deposition, a subset of the 3D-printed PLA scaffolds was subjected to an alkaline surface pre-treatment to enhance surface wettability and promote coating adhesion. The scaffolds were immersed in an aqueous sodium hydroxide (NaOH) solution (1 M) for 2 h under gentle agitation to induce surface activation and thoroughly rinsed with bidistilled water (DDW, AIESI, Hospital Service, Naples, Italy) and dried at room temperature. Untreated PLA scaffolds were used as reference samples. This treatment induces partial hydrolysis of ester bonds at the polymer surface, leading to increased surface roughness and a higher density of polar functional groups, which are beneficial for subsequent ceramic deposition, as widely reported for aliphatic polyesters such as PLA [10,19].

HAp coatings were deposited onto the 3D-printed PLA scaffolds by the sol–gel process. In detail, calcium nitrate tetrahydrate (Ca(NO_3_)_2_·4H_2_O, product code C1396-1KG, Sigma-Aldrich, Tokyo, Japan) and triethyl phosphite (TEP, 98%, P(OEt)_3_, Sigma-Aldrich, Shanghai, China) were used as calcium and phosphate precursors, respectively. Absolute ethanol (C_2_H_6_O, ≥99.9%, VWR International, Leuven, Belgium) and double-distilled water were employed as solvents, while ammonium hydroxide (28.0–30.0% solution, Sigma-Aldrich, Gillingham, UK) was used to adjust the pH. The precursor solutions were mixed under controlled conditions to promote hydrolysis and condensation reactions. Specifically, the sol–gel precursor was prepared by mixing a calcium-based solution (calcium nitrate tetrahydrate in ethanol) and a phosphorus-based solution (triethyl phosphite in ethanol/water (5:1)), maintaining a Ca/P molar ratio of 1.67, suitable for hydroxyapatite formation. Ammonium hydroxide was added to adjust the pH and promote hydrolysis and condensation reactions. Both untreated and NaOH-pre-treated PLA scaffolds were immersed in 8.5 mL of hydroxyapatite sol and gently agitated for 24 h, allowing infiltration throughout the porous structure. Subsequent drying at room temperature until complete solvent evaporation led to gel formation and deposition of a continuous hydroxyapatite layer on the scaffold surface.

### 2.3. Preparation of Dexamethasone-Loaded Polymeric Microparticles

Dexamethasone (DEX, product code PHR1526-1G, Sigma-Aldrich, St. Louis, MO, USA) was selected as a model anti-inflammatory drug and was loaded within polycaprolactone (PCL) microparticles (product code 440744-250G, Mn ≈ 80,000 g·mol^−1^, Sigma-Aldrich, Gillingham, UK) used as polymeric carriers. Microparticles were produced via an emulsion-based solvent evaporation technique. Briefly, PCL and DEX were dissolved in dichloromethane (DCM, anhydrous, ≥99.8%, containing 40–150 ppm amylene as stabilizer; Sigma-Aldrich, Taufkirchen, Germany) to form the dispersed organic phase, consisting of an 8% (*w*/*v*) PCL solution, with DEX added at a 95:5 PCL:DEX weight ratio for drug-loaded formulations. The organic phase was subsequently emulsified into an aqueous phase containing poly(vinyl alcohol) (PVA, 2% *w*/*v*, 87.0–89.0% hydrolyzed, Mw ≈ 13,000–23,000 g·mol^−1^; Acros Organics, Fair Lawn, NJ, USA) as a stabilizing agent, prepared in double-distilled water, under controlled stirring conditions. Solvent evaporation led to the formation of solid drug-loaded microparticles. The obtained microparticles were washed, collected and subsequently dried at room temperature under a ventilated fume hood for approximately 72 h prior to further analyses.

DEX-loaded PCL microparticles (10 mg/mL) were suspended in 0.5 mL of bidistilled water in an Eppendorf tube. The suspension was then applied onto the scaffold by repeated pipetting (Gilson micropipette, Gilson, Inc., Middleton, MI, USA) for a few seconds, in order to promote infiltration into the porous structure. Subsequently, the sample was placed in an oven at 55 °C for 30 min. This thermal treatment was aimed at softening the PCL microparticles, thereby enhancing their adhesion to the internal surfaces of the scaffold.

### 2.4. PCL Microparticle Characterization

The drug content of PCL microparticles was measured using a spectrophotometric method. Absorbance measurements were performed using a UV–Vis spectrophotometer (DU800, Beckman Coulter, Brea, CA, USA) at 240 nm. Precisely measured amounts (3 mg) of microparticles were dissolved in ethanol: acetonitrile (9:1) and the absorbance of the solution obtained was measured at λ = 240 nm and compared with a calibration curve of DEX (0.75–100 µg/mL) [34].

The PCL microparticles’ morphology was examined by scanning electron microscopy (SEM, Zeiss Supra 25, Carl Zeiss AG, Oberkochen, Germany). A droplet of the aqueous phase containing microparticles was placed on an aluminum stub covered with a conductive carbon disk, which was then dried and metallized with a 40 nm thick gold film using a sputter coater (High Resolution Sputter Coater AGB7234 Agar Scientific, Rotherham, UK) (19.30 g/cm3 and 40 mA/s) before analysis by SEM. Observations were performed at 100–1000×.

Microparticle size characterization was performed using ImageJ (Fiji, version 2.14.0/1.54f) on SEM images. Measurements were conducted on empty PCL microparticles and DEX-loaded microparticles prior to integration. Subsequently, the size of microparticles incorporated onto the scaffolds was evaluated using SEM and optical micrographs, analyzing integrated empty microparticles and integrated drug-loaded microparticles.

Chemical characterization of PCL microparticles, both empty and dexamethasone-loaded, was performed by Fourier Transform Infrared Spectroscopy (FTIR)/Attenuated Total Reflection (ATR) mode, using a Nicolet iS5 FT-IR spectrometer (Thermo Fisher Scientific, Waltham, MA, USA) equipped with an iD7 ATR accessory featuring a diamond crystal. For each sample, 32 scans were collected with a spectral resolution of 4 cm^−1^, over a spectral range of 550–4000 cm^−1^. The acquired spectra were processed using OMNIC software (version 9.9.535), applying ATR correction algorithms to allow comparison with conventional transmittance spectra.

The PCL microparticles’ thermal properties were investigated by Differential Scanning Calorimetry (DSC) measurements, using a DSC Q2000-2641 calorimeter (TA Instruments, New Castle, DE, USA). DSC tests were performed on PCL microparticles, both empty and DEX-loaded, in the following conditions: sample weight around 5 mg, temperature range from −60 °C to 100 °C, heating and cooling rates of 10 °C/min, and nitrogen flow rate of 20.0 mL/min. Two thermal cycles, composed of one heating scan and one cooling scan, were carried out.

### 2.5. 3D-Printed Scaffold Characterization

#### 2.5.1. Morphological and Chemical Characterization

Morphological characterization of the 3D-printed scaffolds was carried out to evaluate the porous architecture, surface modifications induced by the coating processes and the distribution of the drug delivery system within the scaffold matrix. Both digital microscopy and SEM were employed to investigate the samples at different length scales. Digital microscopy observations were performed using a Jiusion Endoscope 8 digital microscope (Shenzhen Jiu Sheng Electronic Commerce Co., Ltd., Shenzhen, China), while SEM analyses were carried out using a Supra 25 microscope (Carl Zeiss AG, Oberkochen, Germany) and a Phenom XL G2 system (Thermo Fisher Scientific, Eindhoven, The Netherlands).

Digital microscopy was used for preliminary macroscopic evaluation of scaffold geometry, surface uniformity and overall coating coverage. Moreover, porosity assessment was carried out on digital micrographs of the scaffolds, distinguishing between the outer cylindrical region, characterized by a nominal pore size of 0.4 mm for linear pattern and 1 mm for the triangular pattern, and the inner cylindrical region, with a nominal pore size of 0.3 mm for line pattern and 0.65 mm for triangular. Quantitative image analysis was performed using ImageJ software. A total of around 100 pores were analyzed for the outer cylinder, while 35 pores were measured for the inner cylinder for both patterns.

SEM analysis was subsequently performed to observe the microstructural features of the scaffolds, including pore morphology, coating continuity and adhesion, as well as the presence and distribution of microparticles on the scaffold surface and within the porous network.

Gravimetric analysis was performed to quantitatively evaluate surface modification and hydroxyapatite coating deposition. The mass of the scaffolds was measured before treatment, after NaOH surface pre-treatment, and after hydroxyapatite coating using an analytical balance (Pioneer PA214C, OHAUS Corporation, Parsippany, NJ, USA). The mass variation was calculated to estimate coating deposition efficiency and to support morphological observations.

The characteristic functional groups of both uncoated and HAp-coated PLA scaffold were investigated by FTIR using ATR mode (Nicolet iS5 FT-IR spectrometer, Thermo Fisher Scientific, Waltham, MA, USA). For each sample, 32 scans were collected with a spectral resolution of 4 cm^−1^, over a spectral range of 550–4000 cm^−1^. The acquired spectra were processed using OMNIC software (version 9.9.535), applying ATR correction algorithms to allow comparison with conventional transmittance spectra.

#### 2.5.2. Thermal Characterization

To assess the possible influence of the printing process on the PLA thermal properties, both PLA filament and PLA-printed samples were analyzed through DSC, using a DSC Q2000-2641 calorimeter (TA Instruments, New Castle, DE, USA), in the following conditions: sample weight around 5–10 mg, temperature range from 0 to 200 °C, heating and cooling rates of 10 °C/min, and nitrogen flow rate of 20.0 mL/min. The samples were analyzed under controlled heating and cooling cycles, allowing the determination of thermal transitions such as glass transition temperature (T_g_), melting temperature (T_m_) and crystallization behavior. Two thermal cycles, composed of one heating scan and one cooling scan, were carried out.

The degree of crystallinity was determined according to [35]. Since PLA exhibits a cold crystallization transition, the degree of crystallinity was calculated using Equation (1), where ΔH_m_ represents the enthalpy of fusion, ΔH_cc_ the enthalpy of cold crystallization, and ΔH_m°_ the enthalpy of fusion of the fully crystalline polymer:

(1) $$\chi \left(\%\right)=\frac{\Delta H_{m}-\Delta H_{cc}}{\Delta H_{m}{}^{\circ}}\cdot 10$$

In this study, the value of ΔHm° = 93.7 J/g, as reported by Davachi and Kaffashi [17], was adopted.

#### 2.5.3. Mechanical Testing

Mechanical characterization of the 3D-printed PLA scaffolds was performed to evaluate their suitability for cranial applications in terms of structural stability and load-bearing behavior. Compression tests were selected as the most representative mechanical evaluation, considering the physiological loading conditions experienced by cranial implants.

The tests were carried out using a universal testing machine (MTS Insight, MTS Systems Corportation, Eden Prairie, MN, USA) equipped with a 50 kN load cell, in accordance with the ASTM D695-15 standard for compression testing of rigid plastics [36]. PLA specimens were prepared as parallelepipeds (12.7 × 12.7 × 50.8 mm) and tested in a flatwise configuration, with the 12.7 × 50.8 mm face in contact with the compression platens. Compression was applied at a constant crosshead speed of 1.5 mm/min until the occurrence of structural collapse and subsequent deformation of the scaffold architecture. All tests were performed at room temperature, and six specimens were tested for each infill pattern (Linear and Triangular) to ensure reproducibility.

The mechanical response of the scaffolds was evaluated from the resulting stress–strain curves. Compressive modulus, maximum compressive stress, strain at maximum stress, and toughness were determined and used to compare the mechanical performance of the different scaffold configurations. Mechanical testing was focused on the selected scaffold architectures (Linear and Triangular) to verify that the adopted design strategy provided adequate mechanical performance for potential cranial implant applications.

### 2.6. In Vitro Biological Evaluation

Unless otherwise specified, all chemicals and reagents were purchased from Sigma-Aldrich (Milan, Italy). Both U2OS (human osteosarcoma cell line) cells and 3T3-Swiss fibroblasts (murin embryonic cell line) were cultured at 37 °C in a humidified atmosphere with 5% CO_2_ in Dulbecco’s Modified Eagle Medium (DMEM) supplemented with 10% fetal calf serum (FCS), 500 U/mL penicillin, 10 mg/mL streptomycin, and 20 mM L-glutamine.

#### 2.6.1. Drug Release Assays

To verify the absence of interactions between PCL and DEX, in vitro release studies of DEX-loaded PCL microparticles were performed in ethanol (EtOH) and phosphate-buffered saline (PBS), with drug quantification carried out by UV–Vis spectrophotometry. A fixed amount of microparticles (2 mg) was suspended in 2 mL of the selected medium at 37 °C under stirring at 300 rpm. At predetermined time intervals, 1 mL of solution was withdrawn and replaced with 1 mL of fresh medium to maintain a constant volume. The amount of released drug was determined by measuring the absorbance at 240 nm using a spectrophotometer (BECKMAN Coulter DU800; Brea, CA, USA) and compared with a calibration curve [34].

#### 2.6.2. HPLC Analysis (Drug Penetration in Cells)

DEX quantification in cell lysates was performed by HPLC analysis according to the method described by Mazzinelli et al. (2025) [34]. Briefly, microparticles were deposited onto cells (3T3-Swiss fibroblasts). DEX-loaded microparticles were added to the cell culture medium (10 mL) in a quantity sufficient to provide a DEX concentration of 70 µg/mL. The release of DEX was analyzed after 4 h of incubation with cells at 37 °C though HPLC analysis. As controls, empty microparticles and a non-vehiculated DEX solution at a concentration of 70 µg/mL were used. After incubation, the cells were washed with PBS and lysed by freezing at −80 °C. Cellular lysates were resuspended in 4 mL of water and centrifuged at 15,000 rpm for 10 min at 4 °C. The supernatant was filtered through a 0.22 µm filter and analyzed by HPLC. Analyses were carried out using a C18 reversed-phase column with an isocratic mobile phase consisting of acetonitrile and distilled water (50:50, *v*/*v*), at a flow rate of 0.3 mL/min and detection wavelength of 254 nm. Calibration curves were prepared in the range of 0.625–5 µg/mL, while the Limit of Detection (LOD) and Limit of Quantification (LOQ) were adopted from previously reported values [34]. Recovery studies were performed on spiked cell lysates to assess extraction efficiency.

#### 2.6.3. Cell Proliferation and Differentiation Assays

Cell proliferation and differentiation assays were performed on PLA scaffolds fabricated using either Triangular (T) or Linear (L) infill patterns. The same assays were subsequently conducted on the corresponding hydroxyapatite-coated scaffolds (T + HAp and L + HAp). Prior to use, all samples were sterilized by immersion in 70% (*v*/*v*) ethanol followed by UV irradiation for 30 min.

Cell viability was evaluated by direct contact. Each scaffold was placed in an individual well of a 12-well plate containing DMEM for 24 h. Subsequently, U2OS cells were seeded onto the scaffolds at a density of 20,000 cells/cm^2^ and cultured for 6 and 9 days under the conditions described above. Cells cultured on standard tissue culture plastic were used as reference controls. After the selected incubation periods, cell viability was assessed by the MTT assay according to a previously described protocol [37]. Briefly, a solution of MTT in PBS (5 mg/mL; 100 μL) was added to the culture medium (900 μL) and incubated for 4 h at 37 °C. The resulting intracellular formazan crystals were dissolved in HCl/isopropanol solution (4 × 10^−2^ M, 0.5 mL). The absorbance (ABS) of each sample was measured at 562 nm using an automatic microplate photometer (ELx800; BioTek, Bad Friedrichshall, Germany). Absorbance values were used as a direct indicator of cellular metabolic activity and viability. Each experiment was performed in quadruplicate and repeated three times.

Cytotoxicity induced by DEX-loaded PCL microparticles was evaluated after 24 h of direct contact between 3T3-Swiss fibroblasts and microparticle amounts corresponding to 0.1 mg/mL and 0.05 mg/mL of DEX.

Osteogenic differentiation was evaluated by Alizarin Red S (ARS) staining on all scaffold configurations (T, L, T + HAp and L + HAp). U2OS cells were seeded at a density of 20,000 cells/cm^2^ and cultured either in osteogenic medium supplemented with dexamethasone (10^−8^ M), β-glycerophosphate (10 mM), and ascorbic acid (0.05 mg/mL), or in standard DMEM, used as the non-osteogenic control condition. The assay was performed after 10 and 20 days of incubation [38]. Briefly, cells were fixed with 70% ethanol for 2 h, stained with 1% ARS solution for 15 min, and thoroughly washed with distilled water. The bound dye was subsequently extracted using 10% acetic acid, and 200 µL of the resulting solution was transferred to a 96-well plate for absorbance measurement at 405 nm. Absorbance values were used as an indirect measure of extracellular matrix mineralization and osteogenic differentiation.

#### 2.6.4. Statistical Analysis

Statistical analysis was carried out using one-way analysis of variance (ANOVA) to assess differences among multiple groups, followed by Tukey’s post hoc test for multiple comparisons. Pairwise comparisons were additionally performed using Student’s *t*-test where appropriate. Differences were considered statistically significant at *p* < 0.05.

## 3. Results and Discussion

### 3.1. Scaffold Morphology and Coating Evaluation

The morphology of the 3D-printed PLA scaffolds was analyzed to evaluate the porous architecture of the selected final design and to assess the effects of hydroxyapatite deposition on surface features. The investigation focused on cylindrical scaffolds with hierarchical porosity, identified as the reference configuration for subsequent functionalization steps.

**Figure 2:**
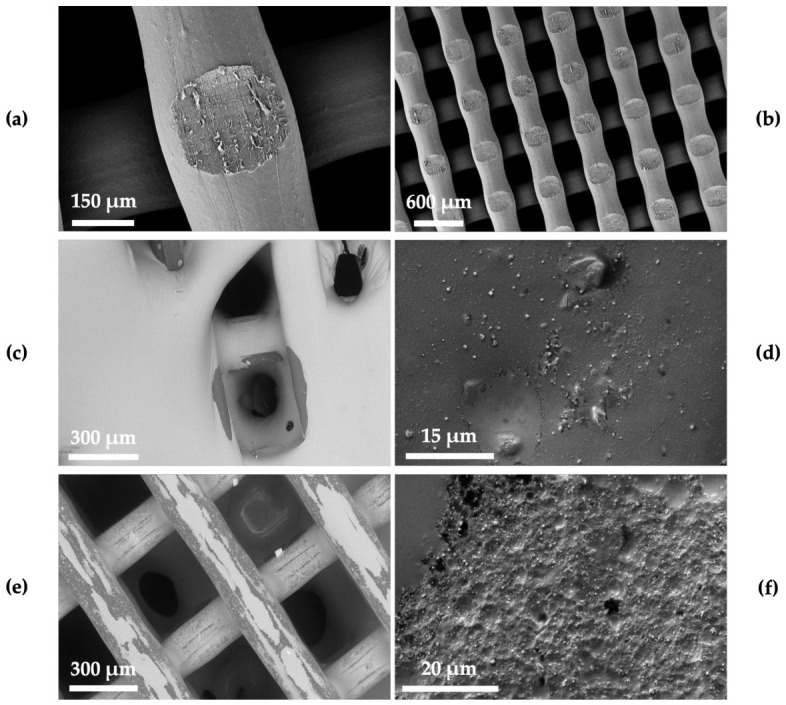
SEM micrographs of 3D-printed PLA scaffolds showing surface morphology and coating features. (**a**,**b**) Bare PLA scaffolds at different magnifications, highlighting the filament surface and porous architecture. (**c**,**d**) Hydroxyapatite-coated scaffolds, without previous NaOH treatment, showing the formation of a continuous ceramic coating uniformly covering the polymeric filaments. (**e**,**f**) Hydroxyapatite-coated scaffolds, after NaOH-pre-treatment, illustrating the effect of surface activation on coating continuity and adhesion.

SEM micrographs of the uncoated scaffolds (Figure 2a,b) evidenced the presence of a well-defined and interconnected porous architecture, consistent with the designed geometry obtained by FDM printing. The printed filaments appeared continuous and regularly arranged, with clearly distinguishable pores distributed throughout the scaffold structure, indicating good printing fidelity and structural reproducibility.

Quantitative image analysis performed on digital micrographs using ImageJ software confirmed that the pore dimensions were consistent with the nominal design values. In particular, the outer cylindrical region exhibited pore sizes of approximately 0.4 mm for the Linear pattern and 1.0 mm for the Triangular pattern, while the inner region showed smaller pores of about 0.3 mm (linear) and 0.65 mm (triangular), in agreement with the designed hierarchical architecture.

After hydroxyapatite deposition via the sol–gel process, clear modifications in surface morphology were observed (Figure 2c,d). The coated scaffolds exhibited the formation of a continuous hydroxyapatite layer covering the polymeric filaments. The ceramic coating appeared homogeneous and uniformly distributed along the scaffold surface, with partial extension into the porous network, resulting in coherent surface coverage throughout the structure.

The effect of alkaline surface pre-treatment was particularly evident in the sol–gel-coated samples (Figure 2e,f). NaOH-pre-treated scaffolds showed improved coating continuity and surface coverage compared to untreated samples, as highlighted by SEM observations, suggesting enhanced interfacial interaction between the polymeric substrate and the ceramic phase [39].

Gravimetric analysis supported the morphological findings and provided quantitative insight into coating efficiency. Scaffolds coated via the sol–gel method exhibited a substantial increase in mass following hydroxyapatite deposition. Specifically, Hap-coated structures showed a weight increase of approximately 35% and 40.1%, in the case of untreated and NaOH-pre-treated samples, respectively. The slight increase detected in the case of NaOH-pretreated samples suggested enhanced ceramic retention promoted by surface activation. The alkaline surface treatment alone resulted in a mass decrease of approximately 10.3%, indicating partial material removal during the NaOH treatment (Table 2). This confirms that the overall mass increase observed in coated samples is primarily associated with hydroxyapatite deposition rather than with the pre-treatment itself.

Overall, the combined SEM and gravimetric analyses demonstrated effective hydroxyapatite deposition on the porous PLA scaffolds and confirmed the beneficial role of NaOH surface pre-treatment in improving coating continuity and retention. Indeed, the differences observed in coating morphology and mass retention can be attributed to interfacial mechanisms governing hydroxyapatite deposition on the PLA substrate. In particular, surface chemistry and deposition dynamics played a crucial role in determining coating effectiveness. Alkaline surface pre-treatment promotes superficial hydrolysis of PLA, leading to the exposure of polar functional groups and increased surface roughness [40]. These modifications are known to enhance surface wettability [41] and favor stronger interactions between the polymeric substrate and the hydroxyapatite precursor during the sol–gel process, facilitating the formation of a more continuous ceramic layer.

### 3.2. Thermal, Spectroscopic and Mechanical Behaviour

Thermal, spectroscopic and mechanical analyses were performed to evaluate the stability of the polymeric material and to assess the effects of scaffold fabrication and surface functionalization on the structural performance of the system.

**Figure 3:**
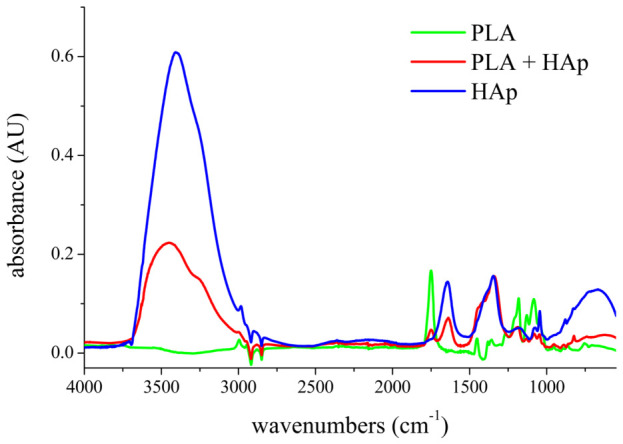
ATR-FTIR spectra of hydroxyapatite sol (blue), bare PLA scaffolds (green) and hydroxyapatite-coated PLA scaffolds obtained by sol–gel deposition (red).

ATR-FTIR spectroscopic analysis was carried out on hydroxyapatite-coated PLA scaffolds to confirm the presence of the ceramic phase and to evaluate the preservation of the polymeric structure after the coating process. Representative ATR-FTIR spectra are reported in Figure 3.

The ATR-FTIR spectrum of the coated scaffolds exhibited the characteristic absorption bands of both PLA and hydroxyapatite, confirming the successful deposition of the ceramic phase onto the polymeric substrate. In particular, the coated samples retain the main PLA features, including the C–H stretching band at approximately 2995 cm^−1^, the ester carbonyl stretching band at ~1750 cm^−1^, and the bands in the 1450–1350 cm^−1^ and 1180 cm^−1^ regions associated with C–H bending and C–O stretching vibrations, respectively [42,43,44].

At the same time, additional absorption bands characteristic of hydroxyapatite are clearly detectable in the coated scaffolds. These include the broad band in the 3700–3000 cm^−1^ range, attributed to O–H stretching vibrations, as well as the phosphate-related bands at ~1045 cm^−1^ (PO_4_^3−^ stretching) and ~668 cm^−1^ (PO_4_^3−^ bending). The presence of these bands, also observed in the spectrum of the HAp sol, confirms the effective incorporation of the ceramic phase onto the scaffold surface. Carbonate-related bands at ~1346 cm^−1^ were also detected, in agreement with literature data for hydroxyapatite-based systems [45].

Importantly, no significant shifts, peak broadening or disappearance of the characteristic PLA absorption bands were observed after the coating process. This indicates that the sol–gel deposition did not induce chemical degradation or undesired modifications of the polymeric matrix. The coexistence of PLA- and HAp-related bands within the same spectrum demonstrates that hydroxyapatite deposition occurred without compromising the chemical integrity of the underlying PLA scaffold. Thermal behavior was investigated by DSC performed on both PLA filament and 3D-printed PLA structure to evaluate possible changes induced by the FDM process. Particular attention was paid to identifying potential changes in thermal properties resulting from additive manufacturing, surface functionalization and microparticle incorporation. The thermal analysis also aimed to verify that the processing conditions adopted for coating deposition and thermal-assisted microparticle incorporation did not induce thermal degradation or undesirable phase transitions in the polymeric materials, thereby preserving their structural integrity and functional performance.

**Figure 4:**
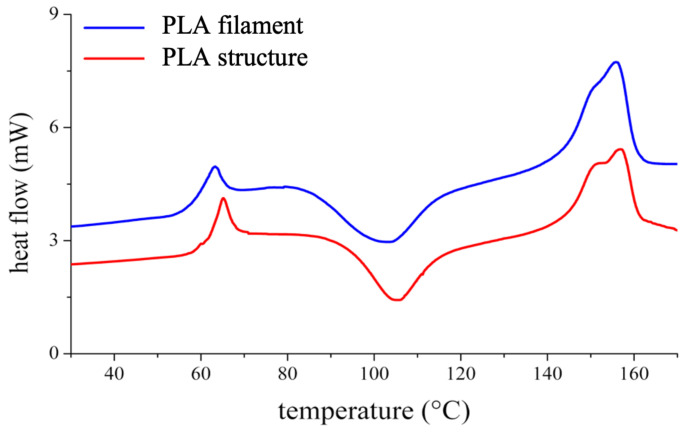
DSC thermograms of PLA filament (blue) and 3D-printed PLA structure (red) recorded during the first heating cycle.

Thermal characterization by DSC was performed on both PLA filament and a 3D-printed PLA structure to evaluate possible changes in thermal behavior induced by the FDM process. The corresponding thermograms of the PLA filament (blue) and the printed PLA structure (red) are reported in Figure 4, while the main thermal parameters extracted from the analysis are summarized in Table 3.

The analysis revealed comparable thermal transitions between the filament and the printed material, suggesting that the FDM process did not induce significant alterations in the overall thermal behavior of PLA. As reported in Table 3, a slight increase in the glass transition temperature (from 60 °C to 63 °C) and in the cold crystallization temperature (from 103 °C to 106 °C) was observed for the printed structure. A modest decrease in crystallization enthalpy was also detected, together with minor variations in the melting behavior.

In particular, the melting peak of the printed PLA structure shows a slight shift and a change in shape compared to the filament, with the onset of peak splitting, as visible in Figure 4. This behavior has been associated with the presence of crystalline populations with different thermal stability, likely resulting from crystal rearrangement or formation during the cold crystallization process [15,46]. A similar, though less pronounced, feature is also observable in the filament thermogram.

Despite these minor variations, the degree of crystallinity of PLA remains essentially unchanged after printing, indicating that the FDM process does not significantly affect the crystalline fraction of the polymer.

**Figure 5:**
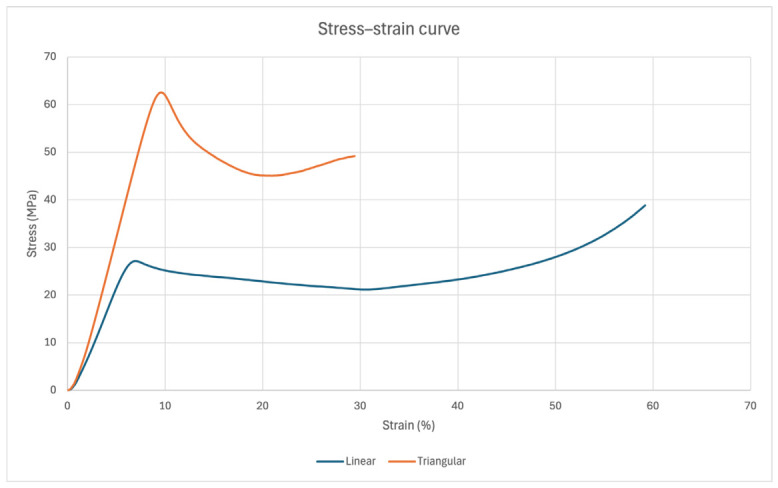
Representative stress–strain curves obtained from compression tests performed on six 3D-printed PLA specimens with Linear and Triangular infill architectures.

Mechanical performance was evaluated through compression tests conducted on PLA specimens to assess the load-bearing capability of the printed material, in accordance with the ASTM D695-15 standard [36]. Representative stress–strain curves obtained for each configuration (Linear and Triangular) are reported in Figure 5.

The results confirmed that both scaffold configurations exhibited a reproducible mechanical response under compressive loading, with limited variability among specimens of the same architecture. As shown in Figure 5, the stress–strain curves were characterized by an initial linear elastic region followed by a pronounced stress peak associated with the onset of structural collapse. Subsequently, a progressive stress decrease was observed, corresponding to the collapse and rearrangement of the internal porous architecture, followed by a gradual increase in stress at higher strain levels due to scaffold densification.

The compression tests allowed the determination of the main mechanical parameters, including maximum compressive stress, strain at maximum stress, compression modulus and toughness. The values obtained by averaging the results from the six specimens tested for each architecture are summarized in Table 4.

These tests provided a baseline evaluation of the mechanical behavior of the polymeric scaffold prior to surface functionalization. Although the measured mechanical properties are lower than those typically reported for bulk PLA, this behavior is consistent with the highly porous nature of the 3D-printed structures. Typical elastic modulus values for compact PLA range from 0.35 to 3.5 GPa [18], whereas significantly reduced values are expected for porous, additively manufactured materials. Moreover, the strain at maximum stress measured in this work falls within the range typically reported for porous additively manufactured PLA structures.

Porosity, internal architecture and loading direction are known to strongly influence the mechanical response of 3D-printed PLA structures. Previous studies have shown that increasing infill density leads to improved mechanical properties [47], while significant variability can be observed among samples with different architectures [19]. The orientation of the applied load relative to the printed layers plays a critical role, as Serra et al. [20] reported up to a threefold difference in elastic modulus depending on loading direction.

In this work, the compressive load was applied in a flatwise configuration. The observed differences between the Linear and Triangular architectures further confirm the strong influence of internal geometry on the mechanical response of porous 3D-printed structures. Finally, although the mechanical behavior was reproducible, the properties of the porous PLA structures remain significantly lower than those of native human bone [48], limiting their applicability to non-load-bearing cranial reconstruction scenarios. Indeed, human cranial bone exhibits compressive strengths typically ranging from approximately 80 to 160 MPa depending on anatomical location, density, and testing methodology [49]. The porous PLA scaffolds developed in this work exhibited lower mechanical performance, which is expected due to their intentionally high porosity and lightweight architecture. The present scaffold was conceived as a proof-of-concept multifunctional platform primarily intended for non-load-bearing cranial applications, where biological integration and local therapeutic functionality represent key design requirements. Future optimization of scaffold architecture, material composition, and reinforcement strategies will be pursued to further improve mechanical performance and approach native bone properties.

Overall, the combined spectroscopic, thermal and mechanical analyses demonstrated that the fabrication process and subsequent surface modification strategies are compatible with the stability of the PLA material, supporting its suitability as a structural substrate for the development of multifunctional scaffolds.

### 3.3. Biological Response of Hydroxyapatite-Coated Scaffolds

The biological response of the hydroxyapatite sol–gel-coated PLA scaffolds was evaluated prior to the integration of the drug delivery system, in accordance with a modular experimental strategy. In this approach, the osteoconductive scaffold and the drug delivery component were initially assessed as independent functional units, in order to isolate their specific contributions and avoid confounding effects.

Two biological assays were performed, namely a cell viability test and a cellular differentiation assay.

**Figure 6:**
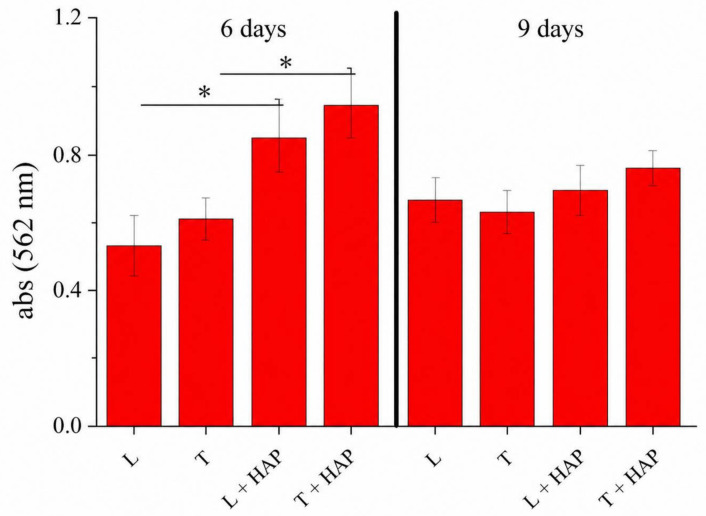
MTT assay results showing cell viability of bare and hydroxyapatite-coated PLA scaffolds at 6 and 9 days, expressed as absorbance at 562 nm. Statistical analysis performed using Tukey’s multiple comparisons test at 6 days compares Linear (L), Triangular (T), and hydroxyapatite-coated samples; * *p* < 0.05.

Cell viability was assessed by means of the MTT assay conducted on both bare and hydroxyapatite-coated PLA scaffolds. The results are reported in Figure 6 as absorbance values, which were used as a direct indicator of cellular metabolic activity and viability. Absorbance values were measured at two different time points, namely 6 and 9 days, to evaluate the temporal evolution of cell proliferation. These time points were selected to monitor the cellular response during the early and intermediate stages of culture on the scaffolds. In particular, the 6-day time point was chosen to assess initial cell attachment and proliferation, whereas the 9-day time point allowed evaluation of the progression of cell growth and scaffold colonization.

At day 6, a significant increase in cell viability was observed for the hydroxyapatite-coated samples compared to the bare PLA scaffolds, indicating a positive effect of the ceramic coating on early-stage cellular response. At day 9, the bare samples showed a slight increase in viability, while the coated samples exhibited a decrease, which can reasonably be attributed to the tendency toward overconfluence after prolonged culture. Overall, these results indicate that hydroxyapatite coating improves the early biological response of the PLA scaffolds and supports cell adhesion and proliferation.

These findings are consistent with previous reports describing enhanced cellular responses on hydroxyapatite-functionalized polymeric scaffolds [22,24]. However, unlike many studies focused exclusively on osteoconductive surface modification, the present work investigates hydroxyapatite functionalization as one component of a broader multifunctional platform intended for the subsequent integration of a therapeutic drug delivery system.

**Figure 7:**
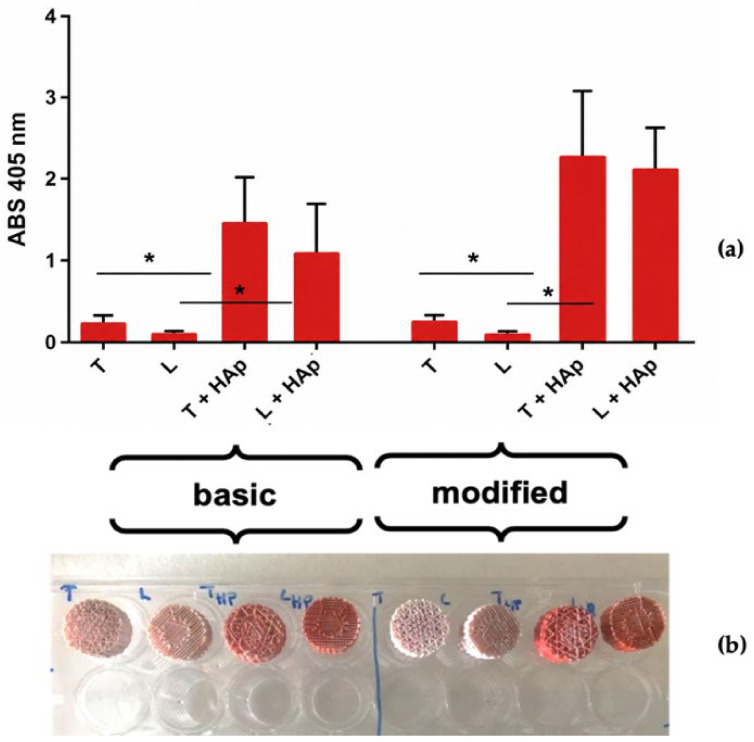
ARS assay results on PLA scaffolds fabricated with Linear (L) and Triangular (T) infill patterns, either uncoated or hydroxyapatite-coated (L + HAp and T + HAp), cultured under basic and osteogenic conditions: (**a**) absorbance values at 405 nm, reported as mean ± standard deviation (* *p* < 0.05); (**b**) representative images of ARS-stained samples, with scaffolds cultured in basic medium (**left**) and osteogenic medium (**right**).

In addition to viability, the osteogenic potential of the scaffolds was investigated through a cellular differentiation assay based on ARS staining. To evaluate both the effect of scaffold architecture and the influence of hydroxyapatite surface functionalization on the biological response, PLA scaffolds fabricated using Triangular (T) and Linear (L) infill patterns were tested before and after hydroxyapatite coating (T + HAp and L + HAp, respectively). The samples were cultured in two different media: a basic culture medium and a modified medium supplemented with growth factors to promote osteogenic differentiation. The results are presented in Figure 7.

The ARS assay revealed a marked increase in mineralization for the hydroxyapatite-coated samples compared to the bare PLA scaffolds, confirming the osteoinductive effect of the hydroxyapatite layer. This effect was particularly evident even in the basic culture medium, suggesting that the hydroxyapatite coating alone is capable of promoting cellular differentiation towards an osteoblastic phenotype.

This observation extends previous evidence on the osteoinductive role of hydroxyapatite-based coatings [23,24], demonstrating that the coated scaffolds are able to promote mineralization even in the absence of osteogenic supplements. Such behavior is particularly attractive for craniofacial tissue engineering applications, where scaffold-driven stimulation of bone regeneration may contribute to improved implant integration.

The presence of growth factors further enhanced this behavior, leading to higher absorbance values in the modified medium.

Overall, these results demonstrate that the hydroxyapatite sol–gel coating significantly improves both cell viability and osteogenic differentiation of the PLA scaffolds, confirming its effectiveness as a bioactive surface modification strategy. These findings provide a solid basis for the subsequent integration of the drug delivery system, whose individual contribution has been independently validated and is discussed in the following section.

### 3.4. Integration and Distribution of the Drug Delivery System

Consistent with the adopted modular strategy, the drug delivery system was first investigated as a standalone component to validate its properties prior to incorporation into the scaffold.

**Figure 8:**
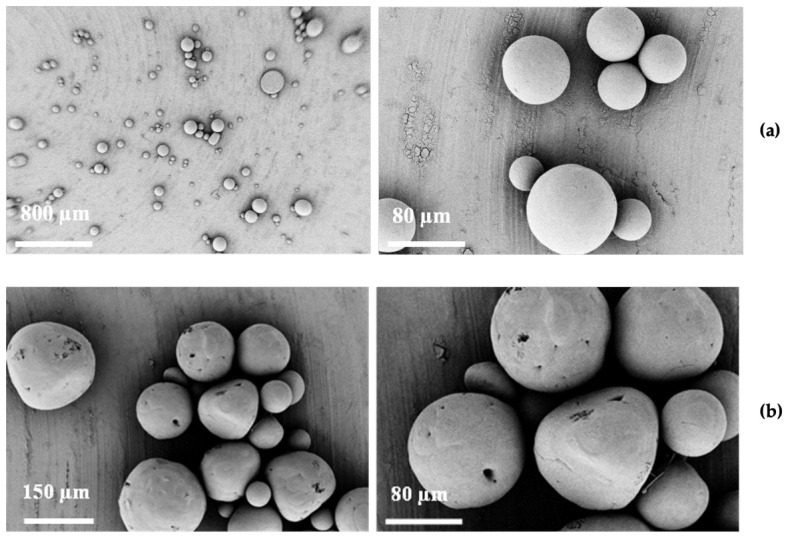
SEM micrographs of dexamethasone-loaded PCL microparticles empty (**a**) and loaded (**b**) before scaffold incorporation, showing spherical morphology and preserved structural integrity at different magnifications.

SEM analysis revealed that the DEX-loaded PCL microparticles were predominantly spherical, with a smooth surface morphology and limited aggregation, indicating good structural integrity before integration (Figure 8). Quantitative image analysis performed on SEM micrographs using ImageJ confirmed these observations, yielding a mean diameter of 73.76 ± 42.40 µm and an aspect ratio of 0.86 ± 0.07. Compared to unloaded microparticles, drug-loaded particles exhibited a slightly larger mean diameter while maintaining a comparable aspect ratio, suggesting that drug incorporation did not significantly alter particle morphology but may have influenced their size distribution.

Drug content analysis performed by UV–Vis spectrophotometry revealed a loading capacity of 25.0 ± 0.5 μg of DEX per mg of PCL microparticles, confirming the successful incorporation of the drug within the polymeric carrier. This result is consistent with the adopted formulation parameters and provides the basis for the subsequent evaluation of drug release behavior.

**Figure 9:**
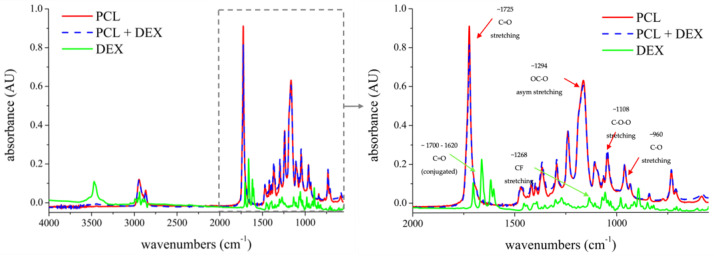
ATR-FTIR spectra of PCL microparticles, both empty (red) and dexamethasone-loaded (blue), compared with pure dexamethasone (green) (wavenumbers range: 500–4000 cm^−1^, (**left**) side; 2000–500 cm^−1^, (**right**) side).

Thermal and structural analyses further supported the stability of the DEX-loaded microparticles. In particular, ATR-FTIR spectroscopy was employed to investigate possible chemical interactions between the polymeric carrier and the encapsulated drug. ATR-FTIR spectra of PCL microparticles, both empty and dexamethasone-loaded, together with the spectrum of pure DEX, are reported in Figure 9. As shown in the spectra, no significant differences were observed between empty and drug-loaded microparticles, and the characteristic absorption bands of dexamethasone were not detectable in the spectrum of the loaded microparticles. Considering that the penetration depth of ATR-FTIR analysis is limited to a few micrometers into the sample surface [50], the absence of dexamethasone-related peaks suggests that the drug is not present on the external surface or in the immediate vicinity of the microparticles.

This behavior has been previously interpreted as an indication of homogeneous drug distribution within the polymeric matrix and as evidence of the absence of chemical bonding between the polymer and the drug [30,51,52].

The main absorption bands observed in the FTIR spectra were assigned to the corresponding functional groups based on literature data [53,54]. In particular, the spectra of PCL microparticles exhibited the characteristic bands of polycaprolactone, including the CH stretching vibrations at approximately 2900 cm^−1^, the carbonyl (C=O) stretching band at about 1725 cm^−1^, CH_2_ bending and wagging vibrations at around 1470 and 1367 cm^−1^, respectively, and the characteristic ester-related vibrations associated with the OC–O and COC groups in the 1294–1242 cm^−1^ region (Figure 9, right side). Additional bands at approximately 1189 cm^−1^ were attributed to CH_2_ deformation vibrations, while the absorptions at around 1108 and 1047 cm^−1^ were associated with C–O–C and ester-related stretching vibrations. The characteristic C–O vibration was also observed at approximately 960 cm^−1^ [53].

The spectrum of dexamethasone showed the characteristic OH stretching band at approximately 3450 cm^−1^, the conjugated carbonyl (C=O) stretching band near 1700 cm^−1^, additional absorptions in the 1660–1620 cm^−1^ region associated with the steroid structure, and the CF stretching vibration at approximately 1268 cm^−1^ [54].

**Figure 10:**
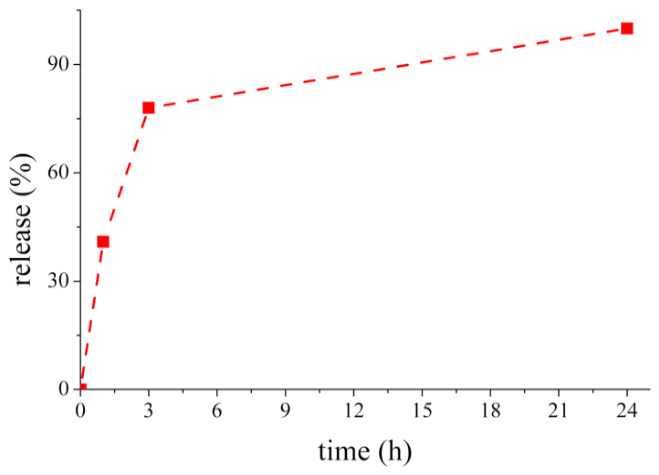
Cumulative release profile of DEX from PCL microparticles measured in ethanol as a preliminary assessment of drug release behavior. Data are presented as mean ± SD (*n* = 3); error bars are smaller than the symbol size and therefore not visible.

The drug release behavior of the microparticles was first evaluated in ethanol to assess DEX diffusion from the polymeric matrix under sink conditions (Figure 10). Drug quantification was performed by UV–Vis spectrophotometry, which represents a suitable and widely adopted analytical method for release studies conducted in simplified media. The loading capacity/total drug content was approximately 25 μg of DEX per mg of PCL microparticles. The release profile showed a rapid initial burst, reaching 40.9% after 1 h and 78% after 3 h, followed by a sustained release phase leading to complete drug release within 24 h. This behavior is characteristic of diffusion-controlled systems and can be attributed to the presence of drug molecules located near or at the particle surface, followed by gradual diffusion from the polymer bulk [55]. Overall, these results confirm the suitability of the microparticles as an efficient drug delivery carrier. The release study was designed as a preliminary proof-of-concept evaluation of the functionality of the microparticle-based drug delivery system rather than as a comprehensive kinetic characterization. Therefore, UV–Vis analysis was considered appropriate under the selected experimental conditions.

**Figure 11:**
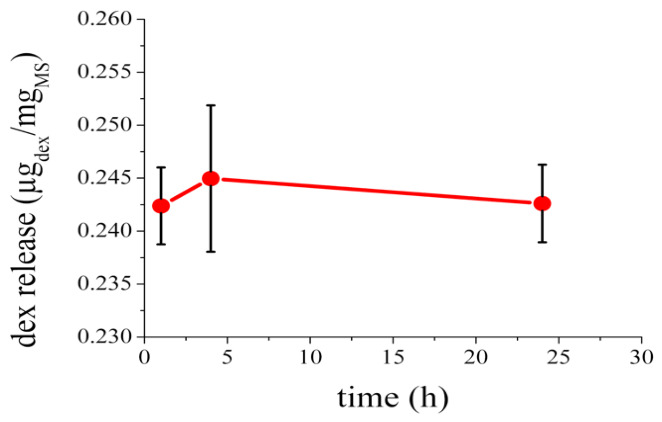
Cumulative amount of dexamethasone released from DEX-loaded PCL microparticles in PBS.

To better approximate physiological conditions, release studies were also performed in PBS (Figure 11). In this case, cumulative DEX release was negligible and rapidly reached a plateau, corresponding to approximately 1% of the total drug load within the first hours. This limited release is consistent with the poor aqueous solubility of DEX [56], indicating that passive diffusion in aqueous media is not sufficient to promote drug release from the PCL matrix. The data are therefore reported as cumulative released amount rather than cumulative percentage release, in order to better visualize the very small variations detected during the experiment.

**Figure 12:**
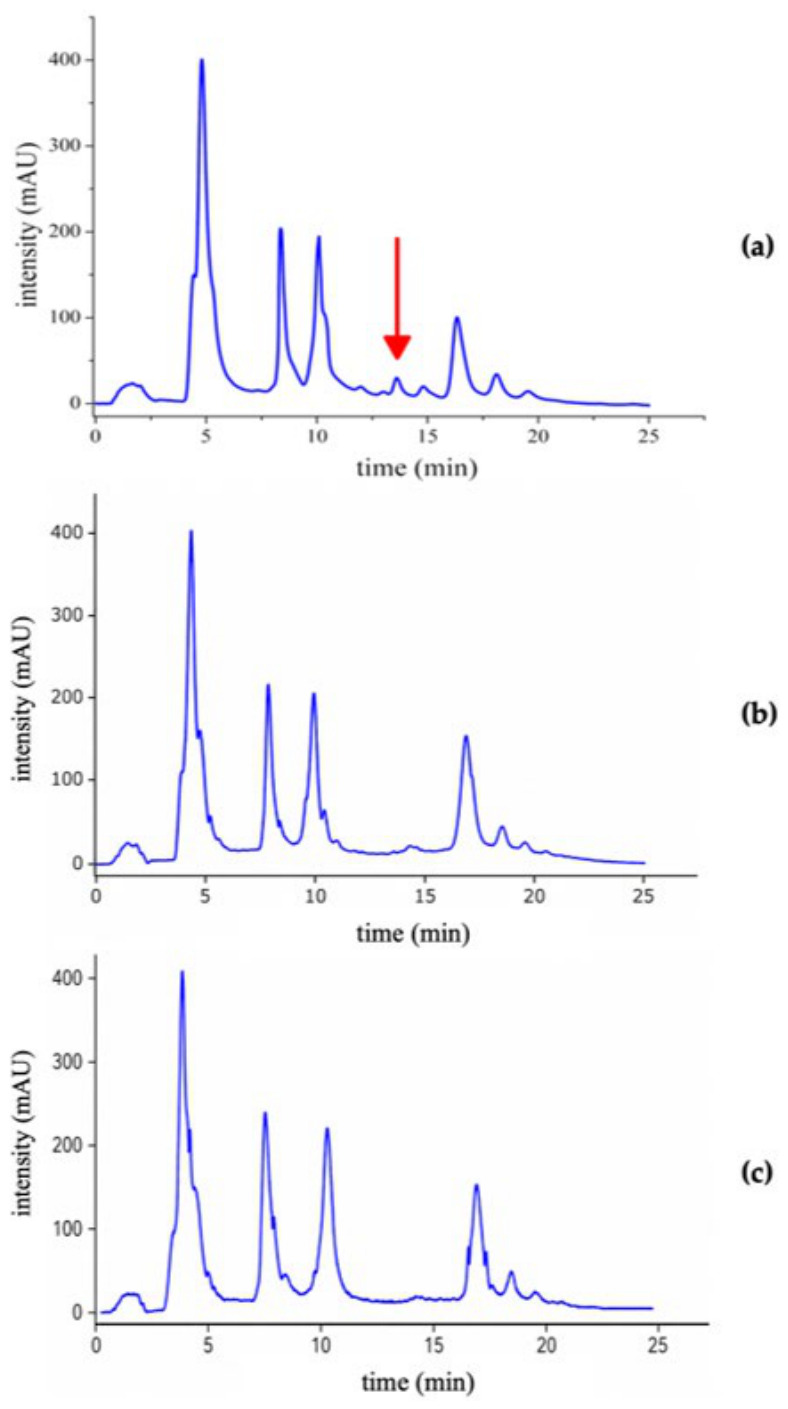
Representative HPLC chromatograms of cell lysates: (**a**) cells exposed to DEX-loaded PCL microparticles, (**b**) cells exposed to empty PCL microparticles, and (**c**) untreated cells. The dexamethasone peak, highlighted by the red arrow at a retention time of 13.60 min, is detected only in lysates from cells exposed to DEX-loaded microparticles, confirming drug internalization.

To overcome the limitations of purely in vitro release tests, an additional experiment was conducted in the presence of living cells. In this case, DEX quantification was performed by HPLC analysis, as the biological matrix of cell lysates required a more selective analytical technique. Following incubation, cellular uptake of DEX was confirmed by HPLC analysis. As shown in Figure 12, a peak at a retention time of 13.60 min was detected exclusively in cell lysates exposed to DEX-loaded microparticles, whereas no corresponding signal was observed in untreated cells or in cells exposed to empty microparticles. Although the measured concentration was above the LOD and below the LOQ, as previously reported for this analytical approach [34], these results demonstrated that drug release occurs in a biologically relevant environment. This suggests that cell-mediated mechanisms or local microenvironmental conditions may facilitate drug availability, which is not captured in standard PBS-based assays.

This finding highlights the limitations of conventional acellular release studies for predicting the biological performance of drug-loaded biomaterials [55]. Unlike many release investigations that rely exclusively on buffer-based assays, the present study combines in vitro release measurements with cellular uptake analysis, providing complementary information on drug availability under biologically relevant conditions.

Overall, this preliminary release study provides evidence of the functionality of the microparticles as a drug delivery system and establishes a reference for their subsequent integration into the scaffold.

**Figure 13:**
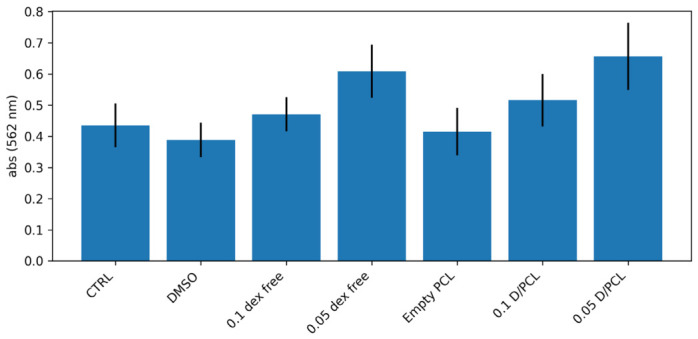
MTT assay performed on 3T3-Swiss fibroblasts after 24 h exposure to empty PCL microparticles, free dexamethasone, and DEX-loaded PCL microparticles at the tested concentrations. Data are presented as absorbance values (mean ± SD), consistently with the other MTT assays reported in this study. Absorbance was used as a direct indicator of cellular metabolic activity. No statistically significant cytotoxic effects were observed under any of the investigated conditions.

The biological response to the microparticles was then evaluated by means of an MTT assay on 3T3 Swiss murine fibroblasts. This cell line was selected in accordance with ISO 10993-5 [57] guidelines for the biological evaluation of biomaterials, as it is widely recommended for cytotoxicity testing. The tested samples included empty PCL microparticles and DEX-loaded microparticles at two different concentrations (0.1 mg/mL and 0.05 mg/mL), selected based on the desired drug dose in contact with the cells and calculated from the total drug loading [58]. For comparison, DMSO alone and free DEX solubilized in DMSO were also included as controls, allowing discrimination between polymer-, solvent-, and drug-related effects. No cytotoxic effects were observed under any of the tested conditions (Figure 13).

**Figure 14:**
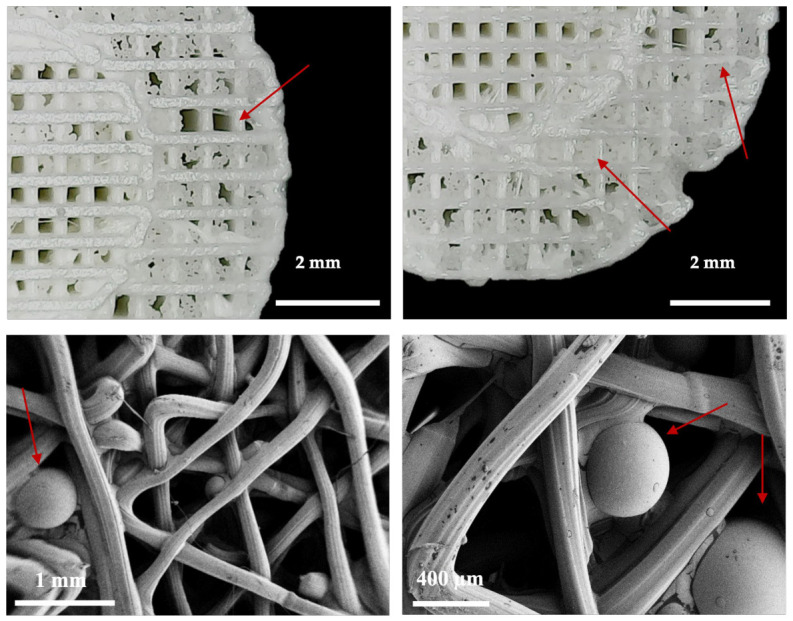
Digital micrographs (**above**) and SEM micrographs (**below**) of scaffolds coated with hydroxyapatite via sol–gel process, showing microparticle distribution within the porous architecture (red arrows).

Following characterization, the microparticles were incorporated into the hydroxyapatite-coated PLA scaffolds using a drop casting thermal-assisted method. Digital observations demonstrated that the microparticles were successfully anchored to the scaffold’s surface and within the porous architecture (Figure 14), while preserving their spherical morphology, as evidence in the related SEM micrograph (Figure 14). A more homogeneous microparticle distribution was observed on scaffolds characterized by continuous hydroxyapatite coatings, whereas heterogeneous ceramic surfaces resulted in localized microparticle accumulation.

Overall, these results indicate that the selected microparticle formulation exhibits suitable morphological, thermal and release properties, and that thermal-assisted incorporation enables effective integration of the drug delivery system into the hydroxyapatite-coated scaffolds.

The long-term fate of the integrated microparticles following scaffold implantation was beyond the scope of the present proof-of-concept study and represents an important aspect for future in vivo investigations.

While numerous studies have reported either osteoconductive PLA/HAp scaffolds or drug-loaded polymeric delivery systems separately [22,24,27,29], the present work demonstrates the feasibility of integrating both functionalities within a single additively manufactured cranial platform through a modular validation strategy. This approach enables independent validation of bioactivity and therapeutic performance prior to their combination into a multifunctional construct.

This proof-of-concept study intentionally focused on demonstrating the feasibility of combining osteoconductive surface functionalization and local anti-inflammatory drug delivery within a single cranial scaffold platform. The adopted design does not aim to fully reproduce the complexity of the clinical scenario, but rather to establish a modular framework enabling independent evaluation of scaffold bioactivity and therapeutic functionality prior to further optimization. In this context, the incorporation of DEX-loaded PCL microparticles should be regarded as a preliminary strategy for local modulation of post-surgical inflammation, while additional studies will be required to assess long-term tissue integration, degradation behavior, and in vivo performance of the fully integrated construct.

## 4. Conclusions

In this study, a multifunctional scaffold platform for cranial reconstruction was developed by integrating additive manufacturing, bioactive surface functionalization and local drug delivery within a single construct. The proposed approach addresses key limitations of conventional cranioplasty solutions by combining structural support, enhanced bioactivity and local modulation of inflammatory responses.

The Fused Deposition Modelling approach enabled the fabrication of lightweight structures with controlled geometry and hierarchical porosity, suitable for bone tissue engineering applications. Hydroxyapatite surface functionalization generated a chemically and structurally stable ceramic interface without compromising the baseline mechanical behavior of the polymeric substrate.

Moreover, the hydroxyapatite coating significantly improved the biological performance of the scaffolds, promoting enhanced cell viability at early time points and inducing osteogenic differentiation, as demonstrated by MTT and ARS assays. These findings confirm the effectiveness of hydroxyapatite as a bioactive surface modification capable of stimulating cellular responses even in the absence of additional biological factors.

The incorporation of a drug delivery system based on DEX-loaded PCL microparticles was successfully achieved through a drop-casting/thermal-assisted strategy, allowing effective integration within the scaffold architecture. The microparticles exhibited suitable morphological stability and drug loading capacity, while release studies demonstrated the ability of the system to provide local drug availability under biologically relevant conditions. HPLC analysis further confirmed DEX uptake by cells, supporting the feasibility of the proposed therapeutic approach.

Overall, the present work demonstrates the feasibility of combining osteoconductive surface functionalization and local anti-inflammatory drug delivery within a single additively manufactured cranial platform. The selection of cranial reconstruction as the target application was intentional, as cranioplasty represents one of the most demanding scenarios for multifunctional biomaterials, requiring the simultaneous integration of structural, biological and therapeutic functionalities.

While further investigations are required to fully characterize drug release from the integrated system and to assess long-term biological performance, including scaffold degradation, tissue integration and the biological response of the fully integrated construct, the proposed modular validation strategy provides a solid foundation for the development of next-generation cranial implants combining regenerative and therapeutic functionalities.

## Figures and Tables

**Table 1:** Printing parameters adopted for the fabrication of cylindrical PLA scaffolds with hierarchical porosity, showing differentiated settings for the inner and outer cylindrical regions.

OUTER CYLINDER—pore size: 0.4 mm (L); 1 mm (T)	
Nozzle = 0.2 mm	Infill Line Direction: [0, 90]
Dimensions: (d = 10 mm; h = 6 mm)	Connect Infill Lines: YES
Layer Height = 0.12 mm	Printing Temperature = 195 °C
Infill Pattern: LINEAR (L); TRIANGULAR (T)	Build Plate temperature = 30 °C
Infill Line Distance = 0.6 mm	Print Speed = 30 mm/s
INNER CYLINDER—pore size: 0.3 mm (L); 0.65 mm (T)	
Dimensions: (d = 5 mm; h = 6 mm)	Infill Pattern: LINES
Infill Line Distance = 0.5 mm	Connect Infill Lines: NO

**Table 2:** Mass variation of PLA scaffolds after alkaline (NaOH) treatment and hydroxyapatite (HAp) coating, expressed as percentage change with respect to the initial mass.

Treatment	Mass Variation
NaOH	−10.3%
HAp	+35%
NaOH + HAp	+40.1%

**Table 3:** Main thermal properties of PLA filament and 3D-printed PLA structure determined by DSC analysis related to the first heating.

Thermal Property	PLA Filament	PLA Printed Structure
Glass transition temperature (T_g_) (°C)	60	63
Cold crystallization temperature (T_cc_) (°C)	103	106
ΔH crystallization (ΔH_c_) (J/g)	18.5	17.5
Melting point (T_m_) (°C)	151–156	151–157
ΔH melting (ΔH_m_) (J/g)	21.4	20.5
Degree of crystallinity (χ) (%)	3.1	3.2

**Table 4:** Mechanical properties of 3D-printed PLA specimens determined by compression testing.

Mechanical Property	Linear	Triangular
Maximum compressive stress (MPa)	27.10 ± 0.26	62.86 ± 0.79
Strain at maximum stress (%)	6.97 ± 0.15	9.56 ± 0.42
Compression modulus (MPa)	400.34 ± 15.01	518 ± 0.42
Toughness (J/m^3^)	0.96 ± 0.02	2.91 ± 0.15

## Data Availability

The raw data supporting the conclusions of this article will be made available by the authors on request.
